# Temozolomide in aggressive and metastatic pituitary tumors: a Brazilian multicenter real-world cohort study

**DOI:** 10.1007/s11102-026-01663-z

**Published:** 2026-03-27

**Authors:** Isabelle Pinheiro Amaro de Magalhães, Camila Regina Pereira Batista de Macedo, Luciana Ansaneli Naves, Mauro Antonio Czepielewski, Isabella Naves Rosa, Lidiana Bandeira de Santana, Tobias Skrebsky de Almeida, Mario Padula, Paula Condé Lamparelli Elias, Margaret de Castro, Leandro Kasuki, Monica Roberto Gadelha, Carolina Garcia Soares Leães Rech, Heraldo Mendes Garmes, Cesar Luiz Boguszewski, Raquel Soares Jallad, Marilena Nakaguma, Maria Candida Barisson Villares Fragoso, Marcio Carlos Machado, Andrea Glezer, Malebranche Berardo Carneiro da Cunha Neto, Olavo Feher, Rafael Loch Batista

**Affiliations:** 1 Neuroendocrinology Unit, Division of Metabolism and Endocrinology, Department of Internal Medicine, Hospital das Clínicas, Faculty of Medicine, University of São Paulo, São Paulo, Brazil; 2 Programa de Pós-Graduação em Ciências Médicas, Faculdade de Medicina, Universidade de Brasília, Brasília, Brazil; 3 Endocrinology Division, Hospital de Clínicas de Porto Alegre, Universidade Federal do Rio Grande do Sul (UFRGS), Porto Alegre, Brazil; 4 Division of Radiology, Hospital das Clínicas, Faculty of Medicine, University of São Paulo, São Paulo, Brazil; 5 Department of Internal Medicine, Division of Endocrinology, Ribeirão Preto Medical School, University of São Paulo, Ribeirão Preto, Brazil; 6 Neuroendocrinology Research Center/Endocrinology Division, Medical School and Hospital Universitário Clementino Fraga Filho, Universidade Federal do Rio de Janeiro, Rio de Janeiro, Brazil; 7 Neuroendocrinology Center, Santa Casa of Porto Alegre, Porto Alegre, Brazil; 8 Service of Endocrinology, Department of Clinical Medicine, Faculty of Medical Sciences, State University of Campinas (Unicamp), Campinas, Brazil; 9 Endocrine Division (SEMPR), Department of Internal Medicine, Federal University of Paraná, Curitiba, Paraná Brazil; 10 Cancer Institute of the State of São Paulo (ICESP), Faculty of Medicine, University of São Paulo, São Paulo, Brazil; 11 Endocrine Genetics Laboratory, LIM-42, University of São Paulo Medical School, Av. Dr. Enéas de Carvalho Aguiar, 255, Cerqueira César, São Paulo, SP 05403-000 Brazil

**Keywords:** Aggressive pituitary adenoma, pituitary carcinoma, Pituitary neuroendocrine tumors, Temozolomide, MGMT

## Abstract

**Purpose:**

To evaluate the real-world efficacy and safety of temozolomide (TMZ) in aggressive and metastatic pituitary neuroendocrine tumors in a Latin American setting, and to address whether TMZ achieves meaningful radiological and biochemical disease control with acceptable toxicity.

**Methods:**

We conducted a retrospective multicenter study across Brazilian reference centers including patients with aggressive/metastatic pituitary adenomas treated with TMZ and followed for ≥ 6 months. The radiological response was assessed via RECIST 1.1. For functioning pituitary adenomas, biochemical response was assessed using prespecified hormonal criteria. Adverse events were collected from medical records.

**Results:**

Thirty patients were included (mean age 29.5 years; 53% female). All the tumors were macroadenomas, and 56% were giant (> 4 cm). Twenty-one pituitary adenomas were functioning and four were metastatic. Ki-67 was > 3% in 73% of the patients. The mean time from diagnosis to TMZ initiation was 102 months. The radiological disease control rate (partial response or stable disease) was 93.3%. Among functioning tumors, the biochemical disease control rate was 81.2%, with an objective biochemical response rate (complete + partial response) of 68.8%. Adverse events, most commonly nausea and myelotoxicity, occurred in 66% of patients.

**Conclusion:**

In this multicenter Brazilian real-world cohort, TMZ provided high radiological and biochemical disease control with an acceptable safety profile, suggesting that TMZ is the preferred first-line systemic chemotherapy for aggressive/metastatic pituitary adenomas after the failure of standard therapies.

## Introduction

Pituitary neuroendocrine tumors are among the most common intracranial neoplasms, accounting for approximately 15% of primary brain tumors [1]. Although the majority of pituitary adenomas follow a benign, slow-growing course, a clinically significant subset exhibits aggressive behavior, characterized by rapid growth and resistance to standard therapies [2–4].

Aggressive pituitary adenomas (APTs) remain a major therapeutic challenge [5]. According to the 2018 European Society of Endocrinology (ESE) guidelines [6], which were reaffirmed by the new 2025 update, APTs are defined not solely by histopathological features but also by clinically relevant progression despite optimized multimodal therapy, including surgery, radiotherapy, and standard medical treatment [2, 7, 8]. The 2025 consensus emphasizes that current histological markers (such as Ki-67, mitotic count, and p53) have limited predictive accuracy, and no validated biomarker that can reliably predict aggressiveness or treatment response currently exists.

Temozolomide (TMZ), an oral DNA-alkylating agent, is currently the only systemic therapy with established efficacy in APTs and metastatic pituitary adenomas [9–12]. It has been recommended as a first-line chemotherapeutic option for APTs and metastatic pituitary adenomas after failure of standard treatments [2]. Its mechanism involves methylation-induced DNA damage at the O6-guanine position, ultimately leading to tumor cell apoptosis [10, 12]. However, the therapeutic response remains highly variable, and complete remission is rare [13]. Although MGMT promoter methylation and low MGMT protein expression have been proposed as predictors of response, their clinical utility remains limited and inconsistent across cohorts [14–16].

Most of the available data on the efficacy and safety of TMZ in APT originate from European centers [17–22]. To date, other populations, particularly those from low- and middle-income countries, have not yet been adequately represented in the literature. This underrepresentation may be clinically relevant, as therapeutic responses could be influenced not only by genetic and epigenetic differences, but also by socioeconomic, healthcare system, and cultural factors that affect diagnosis, treatment access, and follow-up adherence [23, 24].

To address this gap, the present study provides the first comprehensive multicenter analysis of TMZ treatment in APTs conducted in Brazil. By evaluating clinical outcomes, safety profiles, and therapeutic trajectories across multiple national reference centers, this study contributes real-world evidence from an underrepresented population and adds to the global effort to optimize care for these rare and challenging tumors.

## Methods

### Study design and participants

This multicenter retrospective cohort study included eight Brazilian referral centers specializing in pituitary tumors. We screened patients with aggressive pituitary tumors (APTs) or metastatic pituitary adenomas who received temozolomide (TMZ) between 2005 and 2024. The index date was the first day of TMZ cycle 1. Eligible patients had ≥ 6 months of follow-up from TMZ initiation.

Aggressiveness was defined as radiological progression and/or biochemical progression despite conventional therapies (surgery, radiotherapy, and subtype-appropriate medical therapy). Metastatic pituitary adenomas were defined by the presence of craniospinal and/or systemic metastases. In functioning pituitary adenomas, escalation to subtype-specific intensive medical therapy prior to TMZ was recorded as a marker of aggressive/resistant clinical behavior. This included high-dose cabergoline (> 3 mg/week) for prolactinomas; long-acting somatostatin receptor ligands (octreotide, lanreotide, or pasireotide) for acromegaly; and ketoconazole with or without cabergoline for ACTH-secreting tumors, according to local availability. Patients were excluded if they received TMZ for < 3 cycles (or < 3 months) or if severe comorbidities precluded reliable assessment of outcomes. The study was approved by the local ethics committees (with a waiver of informed consent, as applicable), and the data were deidentified before central analysis.

### Data collection

Clinical, pathological, and treatment variables, including tumor subtype, baseline tumor measurements, Ki-67 labeling index, p53 immunohistochemistry, and prior therapies (surgery, radiotherapy, and pituitary-directed medications), were extracted from medical records. TMZ was administered orally on days 1–5 of a 28-day cycle, at 150–200 mg/m²/day, according to institutional protocols. TMZ treatment, treatment interruptions, and the total number of cycles were recorded. Concomitant therapies during TMZ were documented. Radiotherapy, when administered, consists of conventional fractionated external beam radiotherapy (45–54 Gy in standard fractions), radiosurgery or hypofractionated stereotactic radiotherapy according to institutional protocols. No patients underwent concurrent temozolomide and radiotherapy (Stupp protocol).

### Response assessment

Radiological response was assessed via sellar MRI (contrast-enhanced when available) via the longest tumor diameter (1D) and categorized per RECIST 1.1 as complete response (CR), partial response (PR; ≥30% decrease), stable disease (SD; <30% decrease and < 20% increase), or progressive disease (PD; ≥20% increase and/or new lesions/nontarget progression). Imaging assessments followed each center’s routine schedule; when multiple scans were available, the best overall radiological response was recorded. The radiological objective response rate (ORR) was defined as CR + PR, and the radiological disease control rate (DCR) was defined as CR + PR+SD.

The biochemical response was assessed in functioning pituitary adenomas (*n* = 21) and was evaluated in those with paired pre- and on-treatment hormonal data (*n* = 16). It was assessed via 24-h urinary free cortisol (UFC) and, when available, ACTH levels for Cushing disease, age- and sex-adjusted IGF-1 for acromegaly, and serum prolactin for prolactinomas. Given the multicenter nature of the study and heterogeneity in assays and reference ranges across centers, biochemical response categories were defined on the basis of relative change from baseline: complete response (CR; normalization of the available disease-specific biochemical marker), partial response (PR; >20% reduction), stable disease (SD; ≤20% variation), or progressive disease (PD; >20% increase). The biochemical response rate was defined as CR + PR, and biochemical disease control was defined as CR + PR+SD among functioning tumors with available paired biochemical data.

The concordance between radiological and biochemical responses in functioning tumors was assessed via cross-tabulation. Data visualization was performed via heatmaps to display response pairing frequencies and alluvial diagrams to illustrate the flow of patients between response categories.

### Bias

To mitigate bias inherent to retrospective multicenter data, we applied prespecified and standardized definitions for eligibility and outcomes (including RECIST 1D for radiological response and predefined biochemical thresholds for functioning tumors). Data were extracted via a harmonized case-report form and centrally checked for internal consistency. When multiple on-treatment assessments were available, the best overall radiological response was recorded according to uniform criteria. Analyses of biochemical response were restricted to functioning tumors with available data, with no imputation for missing values.

### Safety

Adverse events were collected from medical records and laboratory monitoring during TMZ. Events were categorized as gastrointestinal or hematologic and graded when feasible using the CTCAE criteria; hematologic toxicity was defined on the basis of standardized thresholds for neutropenia and thrombocytopenia.

### Outcomes

The primary outcomes were radiological disease control and overall safety/tolerability. The secondary outcomes included biochemical disease control in functioning tumors and the distribution of radiological and biochemical response categories (CR/PR/SD/PD) during TMZ. Exploratory outcomes included agreement between radiological and biochemical trajectories in functioning pituitary adenomas with available biochemical data.

### Statistical analysis

Categorical variables are presented as n (%) and continuous variables are presented as the mean ± SD or median (IQR), as appropriate. Associations between categorical predictors and response were evaluated via Fisher’s exact test (or the chi-square test when applicable). Ki-67 was analyzed as a continuous variable and dichotomized at 3% and 10%. Continuous Ki-67 values across response groups were compared via the Kruskal–Wallis test with post-hoc Dunn tests when appropriate. A composite “high-proliferation” phenotype (Ki-67 ≥ 10% plus p53 positivity) was explored. Agreement between radiological and biochemical outcomes was evaluated among functioning pituitary adenomas with available hormonal data. For the primary agreement analysis, responses were dichotomized as disease control (CR/PR/SD) versus progression (PD) in each domain, and concordance was summarized using percent agreement and Cohen’s kappa. As a descriptive secondary analysis, cross-tabulations of the original response categories (CR/PR/SD/PD) were reported to characterize discordant patterns. Analyses were two-tailed with α = 0.05 and performed in R (RStudio).

This study is reported in accordance with the STROBE guidelines for cohort studies (Supp Table 1; Supp Fig. 2).

### Ethics approval

This study was approved by the Ethics Committee of the Hospital das Clinicas da Faculdade de Medicina da Universidade de São Paulo (CAAE: 86575324.3.0000.0068).

## Results

**Fig. 1:**
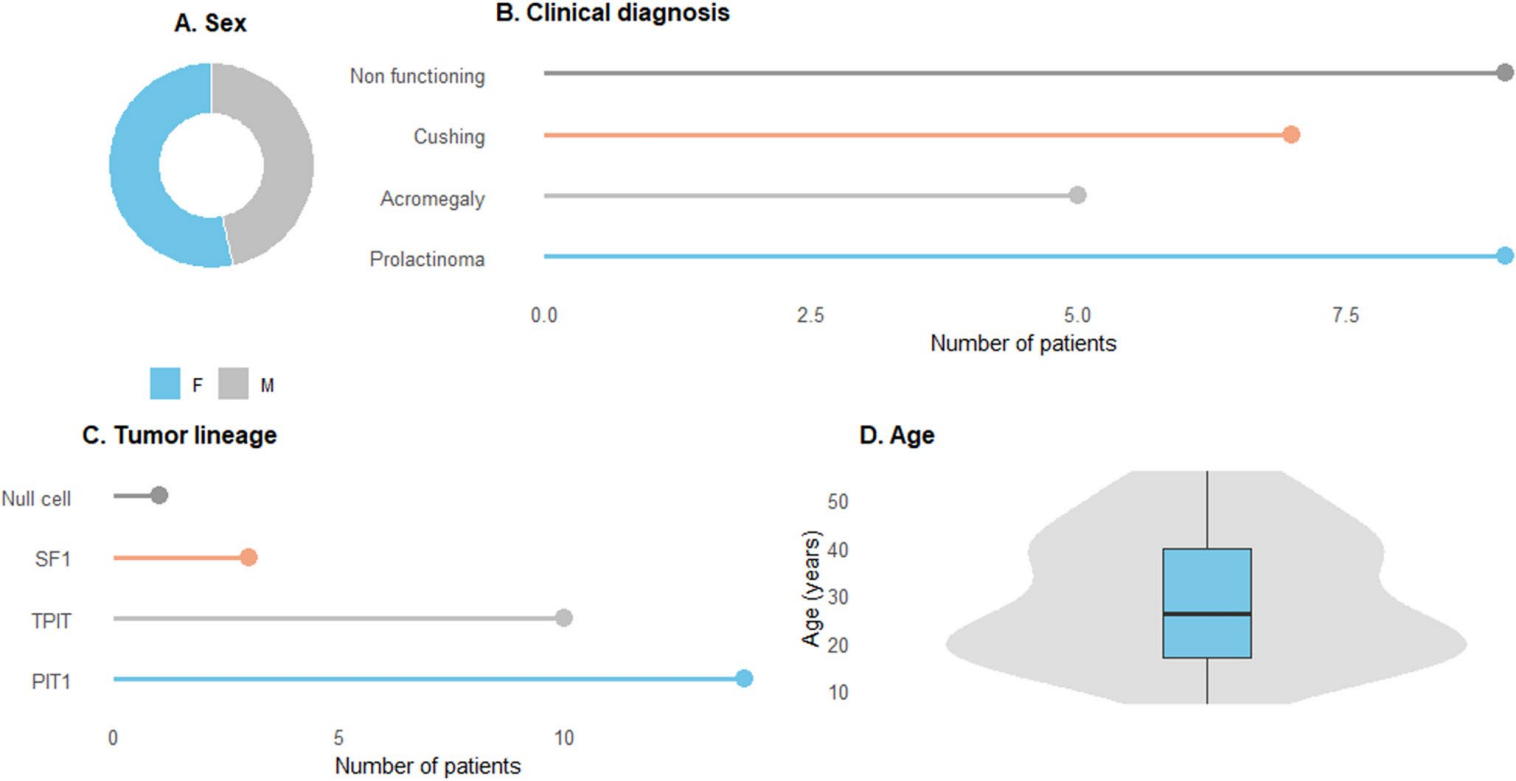
Baseline characteristics of the cohort treated with temozolomide. (**A**) Sex distribution represented as a donut chart. (**B**) Clinical diagnoses at treatment initiation (prolactinoma, acromegaly, Cushing’s disease, and nonfunctioning pituitary adenomas). (**C**) Tumor lineage classification according to the pituitary adenoma lineage (PIT1, TPIT, SF1, and null cells). (**D**) Age distribution shown as combined violin–box plot, illustrating the median, interquartile range, and overall spread

A total of 30 patients with aggressive or metastatic pituitary adenomas were included (Table 1; Suppl Table 1). The mean age at diagnosis was 29.5 years (range 7–56), and 53% of the cohort was female (Table 1; Fig. 1). All patients had macroadenomas, with a mean tumor diameter of 4.2 cm (range 1.2–12.0 cm); 56% were classified as giant tumors (≥ 4 cm). Four patients (13.3%) were diagnosed with metastatic pituitary adenomas, comprising one case each of prolactinoma, nonfunctioning tumor, Cushing’s disease, and acromegaly.

**Table 1:** Clinical characteristics of patients treated with temozolomide (*N* = 30)

Clinical characteristics of patients (*N* = 30)	
Age at diagnosis	29.5 years (7–56 years)
Sex (female/male)	53%/47%
Tumor size at diagnosis	4.2 cm (1.2–12 cm)
Giant Tumors (≥ 4 cm)	56%
Metastatic pituitary adenomas	4/30 patients
Tumor type	
Prolactinoma	8 patients
Non functioning	9 patients
Cushing	8 patients
Acromegaly	5 patients
Treatment before TMZ	
Surgery	2.6 surgeries (1–6)
Radiotherapy (before TMZ)	19 patients
Time between diagnosis and TMZ treatment (median)	54 months (3–336)
Duration of TMZ treatment (median)	6.5 months (3–44)
Immunohistochemical analysis	
Ki-67 (27/30)	13.6% (1–30%)
p53 (22/30)	59% positive

Among the 30 patients, 70% (*n* = 21) had functioning tumors, whereas the remaining 30% (*n* = 9) had nonfunctioning pituitary adenomas (Fig. 1). Among the functioning tumors, prolactinomas were the most commom subtype (*n* = 8; 40%), followed by corticotroph tumors causing Cushing’s disease (*n* = 8; 40%), including Crooke cell pituitary adenoma, in one case, and somatotroph tumors associated with acromegaly (*n* = 5; 25%).

The Ki-67 proliferation index was assessed by immunohistochemical analysis in 27 patients (90%) and was greater than 3% in 22/30 (73%) patients, with a mean Ki-67 index of 13.6%. p53 expression was analyzed in 22 patients and was positive in 59% of them (Table 1).

Before initiating TMZ, patients underwent a mean of 2.6 surgical procedures (range: 1–6). The median time from diagnosis to TMZ initiation was 54 months (range: 3–336; mean 102.0 months), and the median duration of TMZ treatment was 6.5 months (range: 3–44; mean 10.6 months). Twenty-six patients (86.7%) received radiotherapy (RT) at some time points (19 before TMZ and 7 after), whereas four patients did not receive RT. Among the RT-treated patients, most underwent conventional fractionated radiotherapy (*n* = 24), with one patient treated with radiosurgery and one with hypofractionated stereotactic radiotherapy.

The main indications for initiating TMZ therapy were radiological tumor progression (53%, *n* = 16) and worsening biochemical marker levels in functioning tumors (41%, *n* = 13). In a smaller subset (*n* = 3), TMZ was introduced due to metastases Table 2.

**Table 2:** Individual clinical tumor type, previous treatments, temozolomide use and response, and outcomes

ID	Age (years)	Sex	Tumor type		Treatment before TMZ		Temozolomide (months from diagnosis to first prescription/duration of treatment in months)	Tumor size		Response to TMZ treatment		Progression-free survival (months)	Second course TMZ	Deceased
			Clinical tumor type	Tumor lineage	Surgeries (number of procedures)	Radiotherapy		At the diagnosis (cm)	TMZ treatment (before/after -cm)	Radiological response (diameter)	Biochemical response			
1	16	M	Non functioning	TPIT	1	yes	168/5	12	9.3/7.9	SD	-	12	yes	yes
2	29	F	Non functioning	SF1	2	yes	228/10	2.5	5.4/3.7	PR	-	23	no	
3	16	M	Non functioning	NA	2	no	18/3	4.4	4.4/4.8	SD	-	98	no	
4	26	M	Prolactinoma	PIT1	3	yes	84/6	5.0	3.3/3.0	SD	PR	121	no	
5	7	M	Prolactinoma	PIT1	5	yes	120/6	6.5	6.7/7.5	SD	PD	0	no	yes
6	16	F	Prolactinoma	PIT1	2	no	24/3	3.0	4.0/4.5	SD	PR	0	no	
7	45	M	Prolactinoma	PIT1	5	yes	156/6	4.6	5.1/5.2	SD	PR	4	yes	yes
8	16	F	Cushing	TPIT	4	yes	24/18	5.0	3.7/3.5	SD	-	28	yes	
9	26	M	Cushing	TPIT	2	yes	240/4	2.4	1.9/2.4	PD	PD	0	no	
10	15	F	Prolactinoma	PIT1	2	yes	37/9	2.9	2.4/2.0	PD	PD	7	no	
11	39	F	Non functioning	PIT1	2	yes	144/26	2.0	4.8/2.3	PR	-	41	yes	
12	35	F	Cushing	TPIT	3	no	132/6	3.5	3.5/2.5	SD	NA	83	no	
13	22	F	Acromegaly	PIT1	1	yes	48/6	1.2	1.4/0.8	PR	PR	15	no	yes
14	38	M	Cushing	TPIT	6	yes	216/44	4.0	2.3/0.7	PR	SD	44	yes	
15	16	M	Acromegaly	PIT1	3	yes	12/14	6.0	4.0/3.0	SD	NA	12	no	
16	56	F	Cushing	TPIT	2	no	48/15	4.2	4.0/3.0	SD	PR	14	no	
17	40	F	Non functioning	SF1	4	yes	156/3	3.0	4.6/4.7	SD	-	0	no	
18	43	F	Non functioning	SF1	3	yes	96/20	4.0	4.2/4.0	SD	-	120	no	
19	25	F	Acromegaly	PIT1	1	no	3/7	6.3	6.0/3.0	PR	PR	8	no	
20	20	F	Non functioning	Null cell	6	yes	24/6	6.5	5.0/4.0	SD	-	6	no	yes
21	15	M	Prolactinoma	PIT1	3	yes	60/12	4.5	4.3/3.5	SD	PR	60	no	
22	19	M	Acromegaly	PIT1	2	no	38/9	4.8	4.5/3.2	PR	NA	6	no	
23	53	F	Prolactinoma	PIT1	1	no	24/7	3.5	3.6/3.3	SD	PR	7	no	
24	39	M	Acromegaly	PIT1	4	yes	336/21	3.0	4.5/2.5	PR	PR	9	no	yes
25	21	F	Cushing	TPIT	1	no	12/4	2.3	2.9/1.7	PR	SD	10	no	
26	48	F	Cushing	TPIT	1	no	48/6	3.6	3.6/4.1	SD	CR	0	yes	yes
27	53	M	Non functioning	TPIT	3	no	36/12	5.9	5.2/5.5	SD	-	11	no	
28	42	M	Non functioning	NA	2	yes	252/6	NA	3.3/3.3	SD	-	84	no	
29	24	M	Prolactinoma	PIT1	1	yes	48/4	4.6	4.6/4.6	SD	NA	0	no	
30	32	F	Prolactinoma	PIT1	1	no	228/20	1.2	2.2/2.2	SD	PR	30	no	

*F* Female, *M* Male, *CR* Complete response, *PR* Partial response, *SD* Stable disease, *PD* Progressive disease, *NA* not available

**Fig. 2:**
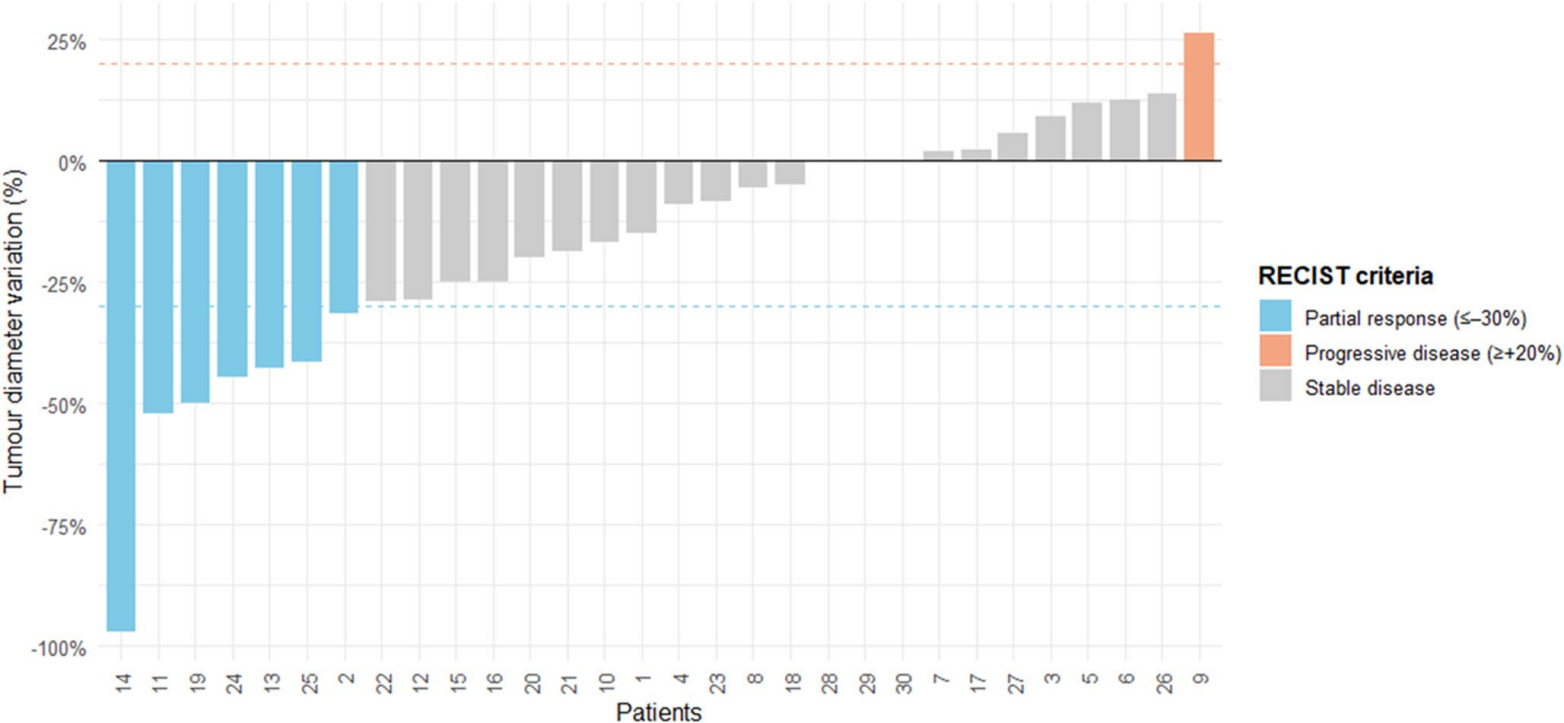
Waterfall plot showing the individual response to temozolomide according to the RECIST criteria. The bars are coloured according to the best RECIST 1.1 response. Each bar represents the percentage variation in tumor diameter relative to baseline for an individual patient. The blue bars indicate partial response (≥ 30% reduction), the gray bars represent stable disease (between –30% and +20%), and the orange bars denote progressive disease (≥ 20% increase). The dashed lines indicate RECIST thresholds for partial response and progression. The bars are coloured according to the best RECIST 1.1 response. Patient 10 was classified as having PD because of a new lesion despite a decrease in the target-lesion diameter

The radiological objective response rate (ORR; CR + PR) was 23.3% (7/30; 95% CI 9.9–42.3) and the radiological disease control rate (DCR; PR + SD) was 93.3% (28/30; 95% CI 82.8–99.9). Progressive disease (PD) occurred in 6.6% (2/30) of the patients (Fig. 2). Notably, one patient (patient 14) with a corticotroph functioning pituitary adenoma exhibited a 97% tumor reduction after six months of TMZ treatment. Although the patient was considered a partial responder, the magnitude of tumor shrinkage was remarkably significant. Additionally, a Crooke cell pituitary adenoma achieved stable disease under TMZ. Taken together, 93.3% of patients (*n* = 28) achieved disease control (SD + PR) during TMZ treatment.

The response profiles to treatment were remarkably similar between functioning and nonfunctioning pituitary adenomas. The rates of partial response were nearly identical (22.2% vs. 23.8%, respectively; *p* = NS), and the proportion of patients who achieved stable disease was comparable (77.8% vs. 66.7%; *p* = NS).

The efficacy of TMZ varied significantly among functioning pituitary adenomas (*p* = 0.012). Prolactinomas (*n* = 8) demonstrated uniform radiological stability (8/8, 100%) without measurable tumor regression; however, biochemically, partial control was achieved in 87.5% (7/8) of the patients, whereas 12.5% (1/8) showed biochemical progression. Among patients with Cushing’s disease (*n* = 8), one patient exhibited disease progression. Biochemically, 16% (1/6) achieved complete hormonal remission, 16% (1/6) achieved partial improvement, 33% (2/6) remained stable, and 33% (2/6) experienced biochemical progression. The biochemical response rate (CR + PR) was 68.8% (11/16), the biochemical disease control rate (CR + PR+SD) was 81.3% (13/16), and biochemical progression occurred in 18.8% (3/16) of the patients. Overall, acromegaly was associated with the highest rate of radiological response among functioning pituitary adenomas in our cohort.

**Fig. 3:**
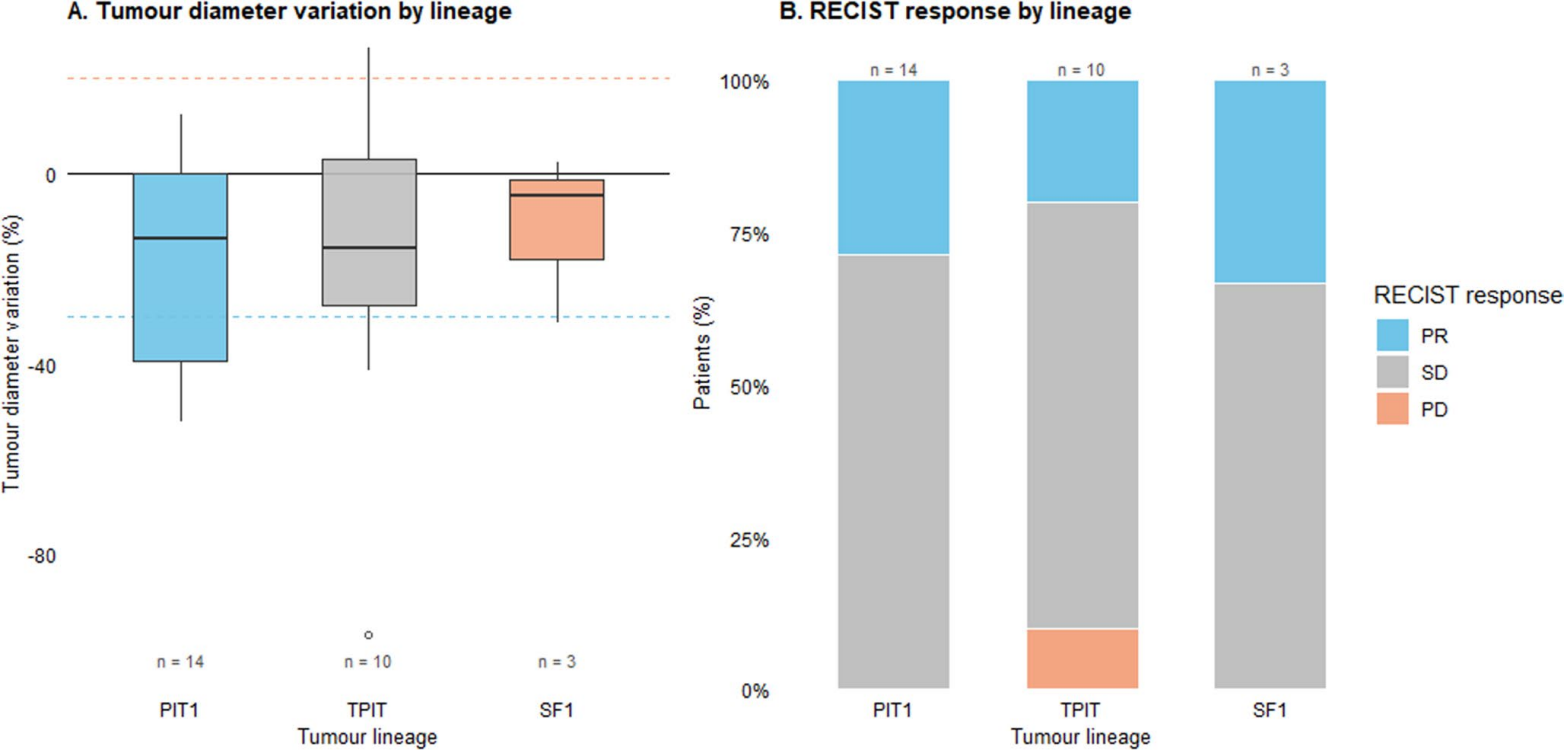
Response to temozolomide according to the pituitary tumor lineage. (**A**) Tumour diameter variation (%) stratified by lineage (PIT1, TPIT, SF1). Boxplots show the median, interquartile range, and full distribution of radiological response; dashed lines represent RECIST cutoffs for partial response (–30%) and progressive disease (+20%). (**B**) RECIST response distribution by lineage, displayed as stacked proportions of partial response (PR), stable disease (SD), and progressive disease (PD). The number of patients per lineage is shown above each bar. PIT1-lineage tumors demonstrated the highest proportion of radiological shrinkage, whereas TPIT and SF1 tumors presented predominantly stable disease

The tumour lineage was available for 27 of the 30 patients (PIT1, *n* = 14; TPI, T *n* = 10; SF1, *n* = 3). The percentage change in the maximal tumour diameter on temozolomide was broadly similar across lineages (median change: PIT1 − 13.8%, TPIT − 15.9%, and SF1 − 4.8%; Fig. 3A). The best RECIST response distributions are shown in Fig. 3B: PIT1 (PR 4/14, SD 10/14), TPIT (PR 2/10, SD 6/10, PD 2/10), and SF1 (PR 1/3, SD 2/3). Two cases of radiological progression occurred in the TPIT group; all PIT1 and SF1 cases achieved radiological disease control (PR/SD).

**Fig. 4:**
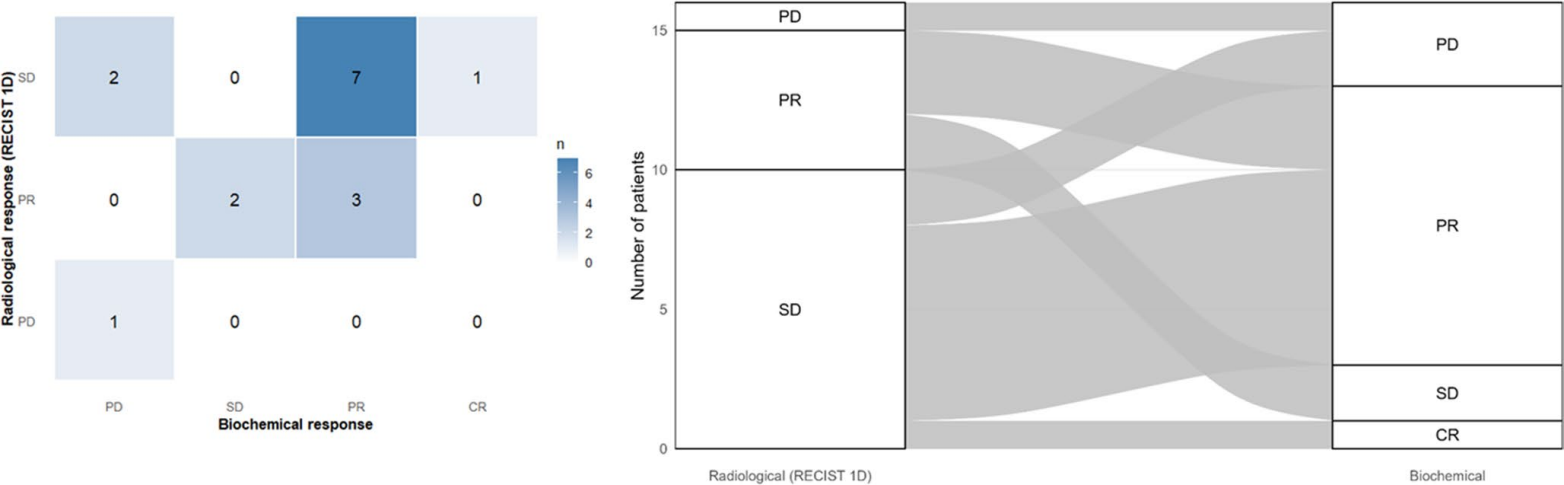
Concordance between radiological and biochemical responses to temozolomide in functioning pituitary adenomas. Left panel: Heatmap cross-tabulation of radiological response by RECIST 1D (PD, PR, SD) versus biochemical response categories (PD, SD, PR, CR) among functioning tumors with available biochemical data (n=16); numbers indicate patients per cell. Right panel: Alluvial diagram depicting flows between radiological and biochemical response categories, with bandwidths proportional to the number of patients in each radiological–biochemical pair. No complete radiological responses were observed. (Abbreviations: PD, Progressive Disease; SD, Stable Disease; PR, Partial Response)

Among functioning tumors with available biochemical data (*n* = 16), the biochemical response (CR + PR) rate was 68.8% (11/16; 95% CI 41.3–89.0), and the biochemical disease control (CR + PR+SD) rate was 81.2% (13/16; 95% CI 54.4–96.0). Radiological and biochemical outcomes were compared at two levels of resolution. When responses were dichotomized as disease control (CR/PR/SD) versus progression (PD), the overall concordance was 87.5% (14/16), with moderate agreement according to Cohen’s kappa (κ = 0.44). When full response categories (CR/PR/SD/PD) were cross-tabulated, category-level concordance was 68.7%, reflecting meaningful dissociation patterns within the disease-control group (Fig. 4). Importantly, biochemical progression occurred in 12.5% of patients (2/16) despite radiological disease control, indicating that biochemical and radiological trajectories may diverge in a subset of patients.

Although the radiological and biochemical responses were generally concordant, distinct patterns of dissociation were observed (Fig. 4). As shown in the heatmap (Fig. 4 - left), two patients (12.5%) presented with biochemical progression despite achieving radiologically stable disease. The alluvial diagram (Fig. 4 - right) further illustrates this divergence, highlighting that radiological stability does not necessarily guarantee hormonal control in a subset of aggressive pituitary adenomas.

Radiological outcomes differed between APTs and metastatic pituitary adenomas. Among the four patients with metastatic disease, two patients (50%) achieved a partial response, whereas the remaining two (50%) experienced disease progression, with no cases of stable disease. In contrast, among the 26 patients with aggressive nonmetastatic tumors, disease stabilization was the most frequent outcome (76,9%).

The potential impact of radiotherapy (RT) timing on the radiological response was evaluated across the cohort. Patients were categorized on the basis of the sequence of RT in relation to the study treatment: RT before, RT after, and no prior RT. Statistical comparison between the two largest groups—RT before (*n* = 19) and RT after (*n* = 7)—revealed no significant association between RT timing and treatment outcome (*p* = 0.65). A high rate of disease stabilization and partial response characterized both groups.

Exploratory analysis of the proliferation marker Ki-67 did not reveal a statistically significant association with the tumor diameter response following TMZ treatment. When evaluated categorically, the chi-square test revealed no significant difference in response proportions among the Ki-67 groups (χ² = 2.47; *p* = 0.65). Similarly, considering the continuous Ki-67%, the Kruskal–Wallis test revealed no significant difference between the response groups (H = 2.17; *p* = 0.34). The Dunn post-hoc test with Bonferroni correction confirmed the absence of relevant pairwise differences (*p* > 0.6 for all comparisons). Although no statistical significance was reached, there was a numerical trend toward higher Ki-67 values in tumors with poorer response: the mean Ki-67 ranged from 12.3 ± 10.0% in stable tumors to 17.8 ± 11.1% in those showing reduction, reaching 20% in progressive cases. When Ki-67 was dichotomized (< 10% vs. ≥ 10%), no statistically significant difference was observed in the tumor response distribution (χ² = 3.11; *p* = 0.21). Nevertheless, all tumors that progressed belonged to the Ki-67 ≥ 10% group.

The p53 expression status, which was assessed by immunohistochemistry and classified as positive or negative, was analyzed with respect to the TMZ response. Within the available cohort, p53 positivity was observed in a greater proportion of tumors (*n* = 14; 64%) than p53 negativity (*n* = 8). The chi-square test did not reveal a statistically significant association between p53 expression and treatment response (*p* = 0.17). However, it is noteworthy that all progressive cases occurred exclusively among p53-positive tumors, whereas none were observed in the p53-negative group.

Since neither Ki-67 nor p53 expression alone reached statistical significance, we explored a composite variable integrating both proliferation and genomic instability markers. Tumors exhibiting Ki-67 ≥ 10% combined with p53 positivity were categorized as highly proliferative lesions. All patients with progressive disease and half of those with partial response were in this high-proliferation category, whereas stable disease predominated among the remaining tumors (Ki-67 < 10% and/or p53-negative).

### Postdiscontinuation outcomes and TMZ rechallenge

Of the 25 patients who discontinued temozolomide (TMZ), 5 underwent TMZ rechallenge. During rechallenge, 3 patients achieved stable disease (SD), 1 achieved a partial response (PR), and 1 had progressive disease (PD) and died. Among the 3 patients with SD during rechallenge, 2 subsequently progressed after rechallenge discontinuation and later died.

Among the remaining 20 patients who did not undergo rechallenge, 4 remained stable after TMZ discontinuation, whereas 8 progressed after stopping TMZ despite having achieved disease stabilization while on treatment. In addition, 4 patients died in this subgroup: at the last TMZ cycle, 2 had SD, and 2 had PR. Overall, 2 deaths occurred during TMZ treatment (both related to tumor compression), and 2 occurred after TMZ discontinuation (due to disease progression).

Among patients who discontinued TMZ, the time from the last TMZ cycle to the next tumor-directed treatment was available for *n* = 10, with a median of 7.5 months (range 0–60; mean 14.9 months). Patients who progressed during TMZ and those who did not discontinue TMZ were not eligible for this interval.

Treatment was generally well-tolerated, with 66.6% (20/30) of patients experiencing at least one adverse event. The majority were classified as grade 1 according to the CTCAE criteria [25]. The most frequently reported events were gastrointestinal, with nausea affecting 70% of patients who reported any adverse events, followed by vomiting in 20% of patients. Hematological toxicity (myelotoxicity) was observed in 45% of these patients. Other nonhematological events included asthenia and headache (15% each), whereas cutaneous reactions were infrequent (5%). Notably, 33% of the cohort reported no treatment-related adverse events.

The long-term tolerability of the treatment regimen was assessed by analyzing treatment discontinuation due to adverse events. Dose reduction was required in patients with grade 3 myelotoxicity, and treatment was discontinued in four patients (13.3%), due to grade 4 myelotoxicity, one due to grade 3 myelotoxicity, and one due to grade 3 asthenia. No treatment-related deaths were reported.

The discontinuation rate was 5% (1/20) in patients treated for less than 12 months, whereas it was 30% (3/10) in those treated for more than 12 months, suggesting that cumulative toxicity may become a more prominent factor influencing treatment feasibility over extended durations.

## Discussion

This Brazilian multicenter cohort provides the largest Latin American dataset to date on the use of TMZ as a single therapy in aggressive and metastatic pituitary adenomas. The radiological disease control rate of 93.3%, including 23.3% partial response and 70% stable disease, supports the activity of TMZ in this setting. While no complete responses were observed, this finding is consistent with the broader literature, in which complete radiological responses are uncommon. Recent meta-analyses have reported partial response and stable disease as the most frequent radiological outcomes (32–33% each), whereas complete response is rare (0.6–4%), and progressive disease remains common (29%) [2, 26]. However, direct cross-study comparisons should be interpreted cautiously because retrospective cohorts differ in patient and tumor characteristics, prior treatments, and response assessment timing, and standardized follow-up protocols are lacking.

Compared with previously published studies, our cohort from the Brazilian multicenter study demonstrated distinct demographic characteristics. With a mean age of 29.5 years, the Brazilian participants were notably younger than those in other cohorts [13, 18, 27, 28], whose mean ages ranged from 40.5 to 58.3 years. Additionally, a greater proportion of women was observed in the Brazilian cohort (53%), whereas the other studies predominantly included male participants, with rates reaching up to 87.5% [13, 18, 27, 28]. These findings suggest potential differences in the epidemiological profile or inclusion criteria across studies, which should be taken into account when interpreting and comparing the results.

Importantly, our results offer novel insights when patients are stratified by tumor subtype, size, functionality, and aggressiveness. Patients with acromegaly exhibit a notably high rate of partial radiological response and no disease progression, a finding that may reflect a distinct somatotroph tumor biology or increased sensitivity to alkylating agents. In contrast, patients with prolactinomas and Cushing’s disease experienced disease progression, suggesting possible lineage-related differences in TMZ responsiveness, which merits further investigation. Within the corticotroph spectrum, Crooke cell pituitary adenomas are a high-risk variants that are often associated with aggressive behavior [29, 30]. The stable disease observed in the Crooke cell population in our cohort suggests that even histologically aggressive subtypes may retain clinically relevant chemosensitivity to temozolomide. Metastatic pituitary adenomas exhibited a dichotomous pattern. Although the small sample precludes definitive conclusions, the data highlight the therapeutic challenge posed by metastatic pituitary adenomas and the need for alternative strategies in this subgroup.

The unpredictable clinical behavior of APT is partly explained by the absence of robust prognostic markers at diagnosis. In our series, Ki-67 and p53 expression alone did not reach statistical significance in predicting the tumor response, corroborating previous evidence that conventional proliferation markers may be insufficient when evaluated independently [31, 32].

Although MGMT expression and promoter methylation have been proposed as predictors of TMZ responsiveness, their utility remains inconsistent and was not assessed in this cohort [22, 33, 34]. We did not assess MGMT expression in our series, which represents a limitation given the role of this DNA repair enzyme in counteracting TMZ-induced cytotoxicity. However, the predictive value of the MGMT status remains inconsistent across cohorts, and the recent ESE consensus no longer recommends routine MGMT immunohistochemistry prior to a trial of TMZ, mainly owing to technical variability, temporal changes in expression, and the lack of alternative therapeutic options in this setting [2]. Thus, while MGMT remains of scientific interest, its clinical utility is still limited, underscoring the need to identify more reliable biomarkers of TMZ response and resistance in APT.

The biochemical responses of functioning tumors are heterogeneous. Although limited by sample size, radiological and hormonal responses may not always correlate, and endocrine control should be independently monitored in clinical practice. A hormonal response, even in the absence of a complete radiological response, may hold substantial clinical significance by contributing to morbidity reduction. The biochemical response rates to TMZ in functioning tumors range widely from 19% to 100% [13, 18, 20, 21, 28, 35–40]. In our cohort, partial biochemical response was most common in prolactinomas and acromegaly, whereas Cushing’s disease displayed the broadest spectrum of biochemical response. Taken together, our results underscore the need for harmonized definitions of biochemical response to enable comparability across studies. Consistent with previous observations, biochemical control in functioning pituitary adenomas was variable and did not always parallel the radiological response. Notably, a recent single-center study identified early initiation of TMZ and low MGMT expression as independent predictors of favorable outcomes, particularly in functional tumors [41]. Nevertheless, many studies have traditionally focused on radiological outcomes as the primary endpoint, relegating hormonal response to a secondary outcome [2, 13, 35]. The literature shows considerable variability in the definition of hormonal response. While some studies define response as a reduction exceeding 50% in circulating hormone levels [13, 34], others adopt a stratification approach analogous to the RECIST criteria, since biochemical response is defined as complete normalization of hormone levels; partial response corresponds to a reduction exceeding 20%, stable disease is defined as a fluctuation within ± 20%, and progressive disease is defined as an increase of more than 20% in hormone levels [21, 42]. We used the latter approach for our evaluations.

An extensive survey demonstrated that clinically functioning tumors were more likely to respond to TMZ than nonfunctioning tumors were, independent of the MGMT status [21]. In our series, however, our radiological response rates were similar between functioning and nonfunctioning tumors, with both partial response (23.8% vs. 22.2%, respectively) and radiological disease control, including partial response or stabilization (90.4% vs. 100%). Therefore, functional status alone may not consistently discriminate responsiveness in real-world practice. This apparent discrepancy could reflect differences in patient selection, the timing of TMZ initiation, or referral bias across cohorts. In addition to these differences, one criterion for the definition of aggressiveness in pituitary tumors relies on resistance to standard therapies, which is more difficult to apply to nonfunctioning tumors. In functioning tumors, medical therapies such as cabergoline or somatostatin analogs provide a clear benchmark of resistance, whereas in nonfunctioning tumors, standard therapy is essentially surgical and adjuvant radiotherapy, with limited medical alternatives. Although cabergoline has been investigated in nonfunctioning pituitary tumors, with small series reporting disease stabilization and modest tumor shrinkage, it has not been systematically evaluated in a subset of aggressive nonfunctioning pituitary adenomas [43–46]. These conceptual differences underscore the challenges in establishing uniform criteria for aggressiveness across different tumor subtypes. Importantly, while functioning tumors allow for the additional endpoint of biochemical control, our data reinforce that radiological assessment remains the critical measure of efficacy across all subtypes.

Radiological and biochemical endpoints are not fully interchangeable in functioning tumors. Although overall concordance was high, a clinically relevant discordance emerged: biochemical progression occurred in 12.5% of patients despite radiological disease control. This finding supports independent monitoring of imaging and biochemical markers during TMZ, particularly in functioning pituitary adenomas where biochemical changes may directly translate into morbidity. The moderate kappa coefficient observed despite high percent agreement likely reflects the highly unbalanced distribution of radiological outcomes (predominance of PR/SD and rare radiological PD).

Padovan et al., in a systematic review focused on temozolomide (TMZ) discontinuation, reported progression after TMZ withdrawal in 13/52 patients (25%) and emphasized the absence of prospective data to define the optimal treatment duration; they also noted that, in the absence of prospective studies, most investigators tended to extend TMZ therapy to 12 months when effective. In the same discussion, they stated that there is no international consensus on treatment choice at recurrence after an effective TMZ course, although a second TMZ challenge seems reasonable, and that the reported response rate to rechallenge is more or less halved in the literature. Consistent with this clinical uncertainty, loss of disease control after TMZ discontinuation was frequent in our cohort, including among patients who achieved SD or PR while on treatment. TMZ rechallenge provided temporary disease control in some patients (SD/PR), but responses were not uniformly durable, and progression still occurred in a subset. These findings may be clinically relevant when considering treatment duration and postdiscontinuation surveillance, but they should be interpreted cautiously given the small sample size, retrospective design, and clinical heterogeneity.

The safety profile of TMZ in our cohort was globally acceptable, with adverse events documented in 66% of patients, predominantly grade 1. Nausea and myelotoxicity were the most frequent toxicities, and treatment discontinuation was required in four patients (13%) because of grade 3–4 events, with no deaths related to toxicity. These findings align with the broader literature synthesized in the recent international consensus on APT and metastatic pituitary adenomas [2, 13, 47]. Discontinuation rates due to adverse events range from 6% to 15%, most often driven by pervasive fatigue, nausea, or cytopenias, while rare but severe complications such as aplastic anemia, hepatotoxicity, or secondary hematologic malignancies have been reported [2, 13, 47]. Compared with these data, our series underscores two complementary perspectives: first, the burden of mild adverse effects is common and expected in real-world settings; second, that clinically significant hematological toxicity requiring drug withdrawal is not negligible and should be anticipated in routine practice. Importantly, while we did not observe the rare life-threatening complications highlighted in the consensus, our findings reinforce the need for vigilant hematologic monitoring, as hematologic toxicity remains the leading barrier to treatment continuity in this patient population.

Our study revealed significant heterogeneity in the duration of TMZ treatment, reflecting the current lack of well-defined protocols for the management of APT and metastatic pituitary adenomas. The mean duration of TMZ use was 10.7 months (range, 3–44 months), highlighting the variability in clinical decisions to initiate TMZ. In some cases, treatment was discontinued after six months due to disease stabilization, emphasizing the need for protocols that support both earlier initiation and longer treatment courses. Given that TMZ is most often prescribed following documented disease progression, tumor stabilization should be considered a favorable therapeutic outcome.

According to the 2025 ESE Clinical Practice Guidelines [2], for patients who respond to a first course of TMZ—defined as either partial tumor regression or stabilization after rapid progression in the preceding six months—it is now recommended that therapy be continued for at least 12 months and further guided by efficacy and tolerability. Extending treatment beyond 24 months should be carefully weighed against the potential for cumulative severe toxicity. In line with these recommendations, an Italian real-life study including patients treated for more than 12 continuous cycles reported that prolonged TMZ administration was safe and associated with durable disease control and hormonal improvement, particularly among responsive functioning pituitary adenomas [28].

In our study, 10 patients received TMZ treatment for 12 months or longer. Among them, 81.8% experienced at least one adverse event, most of which was mild in severity. Three cases of myelotoxicity were observed, leading to treatment discontinuation in two patients. The treatment discontinuation rate due to adverse effects was higher in the group that used TMZ for more than 12 months (30% vs. 5%), whereas the myelotoxicity rate was similar between the groups (30% vs. 30%). This suggests that cumulative toxicity and treatment fatigue, rather than hematologic events per se, may limit the long-term tolerability of TMZ in APT.

In the present cohort, despite a tumor control rate of 93.3% with TMZ, mortality was observed in 7/30 patients (23.3%), two due to direct tumor-related complications (mass effect) and five due to disease progression after TMZ withdrawal, despite having initially achieved stable disease (*n* = 3) or partial response (*n* = 2). These findings reinforce that, in the absence of severe adverse effects, maintaining TMZ in patients with a favorable response may be a safe and effective strategy, even when complete radiological remission is not achieved.

### Limitations

This retrospective multicenter cohort reflects real-world practice in Brazilian tertiary referral centers, where temozolomide is typically reserved for highly pretreated aggressive or metastatic pituitary adenomas. Although prespecified response definitions were applied, heterogeneity in treatment timing, imaging schedules, and biochemical assays across centers, along with missingness in key pathology variables (Ki-67/p53) and limited paired biochemical data, may introduce measurement variability and constrain subgroup and concordance analyses. Therefore, the results should be interpreted as descriptive and hypothesis-generating, and extrapolation to earlier-line use or to settings with different monitoring practices should be made cautiously.

Systematic germline or somatic genetic testing was not performed in this retrospective multicenter cohort. Because genetic testing was not uniformly available across participating centers, it was not included as a predefined variable. We acknowledge this as a limitation, particularly for pediatric or young-onset cases, in which an underlying genetic predisposition may be clinically relevant.

## Conclusion

TMZ was effective and generally well tolerated in this Brazilian real-world multicenter cohort, yielding high rates of radiological disease control and a low discontinuation rate. In functioning tumors, biochemical responses are heterogeneous and can differ from imaging outcomes, supporting parallel endocrine and radiological monitoring. Prospective, standardized multicenter studies are needed to validate biomarkers and refine the optimal TMZ timing and duration within individualized therapeutic pathways for aggressive/metastatic pituitary adenomas.

## Data Availability

No datasets were generated or analysed during the current study.
